# Advances in Host–Pathogen Interactions in Tuberculosis: Emerging Strategies for Therapeutic Intervention

**DOI:** 10.3390/ijms26041621

**Published:** 2025-02-14

**Authors:** Mohammad J. Nasiri, Vishwanath Venketaraman

**Affiliations:** 1 Department of Microbiology, School of Medicine, Shahid Beheshti University of Medical Sciences, Tehran 19839-69411, Iran; mj.nasiri@hotmail.com; 2 College of Osteopathic Medicine of the Pacific, Western University of Health Sciences, Pomona, CA 91766-1854, USA

**Keywords:** *Mycobacterium tuberculosis*, virulence factors, host immune mechanisms

## Abstract

Tuberculosis (TB) remains one of the most challenging infectious diseases, with *Mycobacterium tuberculosis* (Mtb) employing sophisticated mechanisms to evade host immunity and establish persistent infections. This review explores recent advances in understanding Mtb’s immune evasion strategies; granuloma dynamics; and emerging immunotherapeutic approaches. Key findings highlight the manipulation of host autophagy; metabolic reprogramming; and cytokine pathways by Mtb to sustain its survival within host cells. Insights into granuloma formation reveal the critical role of bacterial lipids; immune modulation; and hypoxia-driven dormancy in maintaining chronic infection. Innovative therapeutic strategies, including host-directed therapies; epigenetic interventions; and immunomodulators, hold promise for improving TB management and combating drug-resistant strains. Despite these advancements, significant challenges remain, including the development of effective vaccines; addressing latent TB; and ensuring equitable access to novel treatments. The integration of advanced technologies such as artificial intelligence and multi-omics approaches, alongside global collaboration, is essential to overcome these hurdles. This review underscores the importance of a multidisciplinary approach to tackling TB, with the ultimate goal of eradicating this global health threat.

## 1. Introduction

Tuberculosis (TB) remains one of the deadliest infectious diseases, with an estimated 10.8 million global cases in 2023, of which 6.1% occurred among people living with HIV [1]. Despite significant advances in treatment, *Mycobacterium tuberculosis* (Mtb), the causative agent, has developed sophisticated mechanisms to evade host immunity, resulting in chronic infection. The 2024 Global TB Report highlights the persistent challenges in combating TB, noting that, despite a slight reduction in incidence in 2023, TB continues to be the leading infectious killer globally, with deaths only decreasing by 23%, far below the target of a 75% reduction [1].

This ongoing high mortality emphasizes the need for a better understanding of TB pathogenesis and the host–pathogen interactions that allow Mtb to persist. Recent breakthroughs in molecular immunology and advanced technologies are enhancing our comprehension of these interactions, particularly in how the immune system responds to Mtb and how the pathogen manipulates the host environment to establish chronic infections.

This review aims to explore the latest insights into Mtb’s immune evasion strategies, granuloma dynamics, and emerging therapeutic strategies targeting host–pathogen interactions. These developments provide new avenues for tackling TB, which remains a significant global health threat despite ongoing efforts.

## 2. Emerging Mechanisms of Immune Evasion

### 2.1. Phagosome Maturation and Survival

**Figure 1:**
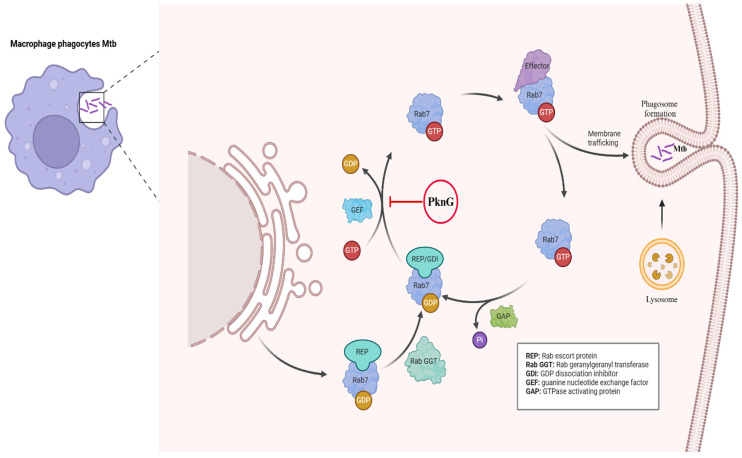
Inhibition of phagosome–lysosome fusion by mycobacterial PknG. PknG interacts with Rab7, a Rab GTPase, and prevents its activation by inhibiting the transition from Rab7-GDP to Rab7-GTP. This disrupts the recruitment of active Rab7-GTP to phagosomes, preventing phagosome–lysosome fusion and enabling Mtb survival within macrophages.

When Mtb is engulfed by alveolar macrophages, it is expected to be degraded within the phagosome. However, Mtb has developed sophisticated strategies to evade this process, utilizing proteins such as protein kinase G (PknG), protein tyrosine phosphatase A (PtpA), and secreted acid phosphatase M (SapM) to survive and replicate within host cells [2,3]. PknG is a serine/threonine protein kinase that plays a crucial role in Mtb pathogenicity and metabolism. It prevents both phagosome–lysosome fusion and autophagosome maturation. PknG achieves this by phosphorylating host proteins involved in vesicle trafficking, blocking bacterial degradation inside macrophages. Specifically, it targets Rab GTPases, particularly RAB7, and inhibits the hydrolysis of RAB7-GTP, preventing the maturation of autophagosomes and blocking the final stages of autophagy (Figure 1) [2,3].

PtpA, a secreted tyrosine phosphatase, disrupts phagosome maturation by targeting the host protein VPS33B, a regulator of membrane fusion in the endocytic pathway [4,5]. By dephosphorylating VPS33B, PtpA blocks the normal progression of the phagosome into a degradative phagolysosome. Additionally, PtpA prevents the assembly of the V-ATPase proton pump, which is responsible for acidifying the phagosome. By binding to the pump’s H subunit, PtpA reduces proton transport, stopping the acidification that is necessary for bacterial destruction [6].

SapM, another secreted phosphatase, alters the host’s phosphoinositide metabolism. Phagolysosome formation requires phosphatidylinositol 3-phosphate (PI3P) on the phagosome membrane, which recruits proteins like EEA1 and Hrs that are essential for phagosome maturation. SapM dephosphorylates PI3P, preventing these crucial proteins from being recruited and blocking phagosome–lysosome fusion [7,8,9,10,11].

### 2.2. Alternative Mechanisms of Phagosomal Escape

Mtb typically blocks phagosome–lysosome fusion through specific virulence factors. However, even in the absence of these factors, such as in Mtb mutants lacking *sapM* or *pknG*, infection can still occur, albeit less efficiently [12,13]. This suggests that Mtb has redundant mechanisms to compensate for the loss of key virulence factors. One well-documented mechanism is phagosomal rupture, where Mtb escapes the phagosome to the cytosol, avoiding lysosomal degradation [12,13,14,15]. This is mediated by bacterial effectors that disrupt the phagosomal membrane, enabling Mtb to replicate in a more favorable environment [12,13,14,15]. For instance, Mtb’s cell wall lipids, like phthiocerol dimycocerosates (PDIMs), help ensure survival within macrophages by preventing phagosome–lysosome fusion, even in the absence of other virulence factors [13]. Additionally, Mtb promotes lipid droplet formation in host macrophages by using urease C (UreC) to inhibit DNA repair and activate the cGAS/STING pathway, which in turn upregulates IFN-β and SR-A1, supporting bacterial replication. Inhibiting UreC impairs Mtb growth, revealing a novel immune evasion strategy [16]. Beyond these mechanisms, recent studies have identified additional strategies employed by Mtb to evade host defenses, including autophagosome maturation disruption and the manipulation of host metabolism [17,18,19]. Despite significant advances, much remains unknown about the specific molecular pathways that allow Mtb to evade phagosome–lysosome fusion. Future studies are needed to elucidate how different strains of Mtb exploit varying strategies and to identify potential therapeutic targets within these mechanisms.

### 2.3. Autophagosome Maturation and Survival

Once Mtb has successfully escaped the phagosome or even while still inside it, it continues to block autophagy, another important host defense mechanism. Autophagy is a process that targets damaged organelles and intracellular pathogens for degradation, but Mtb has developed multiple strategies to prevent this process and ensure its survival within the host [20,21,22,23,24,25] (Table 1).

A recent study found that Mtb’s virulence factor, PDIM, plays a significant role in helping Mtb survive within macrophages by resisting autophagy [26]. PDIM was shown to inhibit LC3-associated phagocytosis (LAP) by blocking the recruitment of NADPH oxidase to the phagosome, which prevents the degradation of Mtb within macrophages. Interestingly, while PDIM was not necessary for Mtb survival in alveolar macrophages during acute infection, it became essential for survival in non-alveolar macrophages in an autophagy-dependent manner [26]. These findings suggest that the bacteria’s ability to manipulate macrophage responses is crucial for its persistence.

Mtb also employs the effector protein EsxH to interfere with the Endosomal Sorting Complex Required for Transport (ESCRT) machinery, particularly targeting the VPS4 subunit. This interaction prevents autophagosome–lysosome fusion, halting the autophagic process and allowing Mtb to evade degradation within macrophages. By disrupting ESCRT functionality, EsxH enables the bacteria to survive [27].

EsxA (ESAT-6), another Mtb effector, forms pores in the lysosomal membrane, leading to dysfunction and impaired autophagic degradation. This effector also activates the cGAS-STING pathway, resulting in increased type I interferon production, which suppresses autophagy and favors bacterial survival [28,29,30]. High-resolution imaging studies have shown that EsxA’s activity near autophagolysosomal compartments correlates with impaired lysosomal acidification, allowing the bacteria to persist within the host [28,29,30].

**Figure 2:**
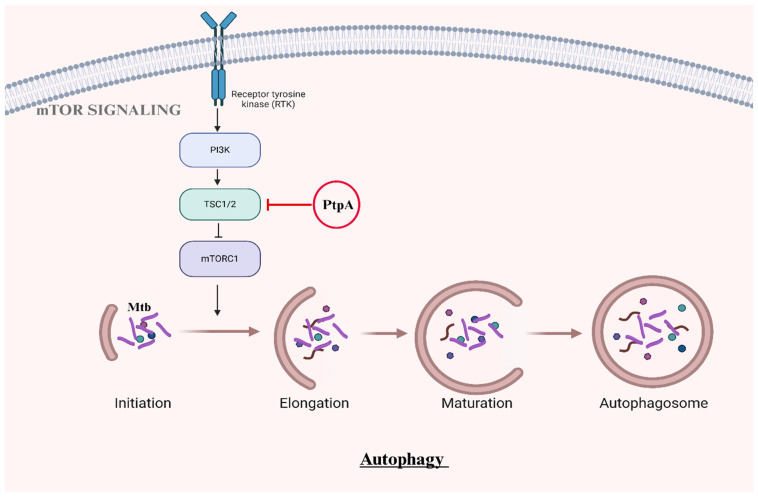
Manipulation of mTORC1 by Mtb to suppress autophagy. Mtb effector protein PtpA dephosphorylates TSC2, a component of the TSC complex that normally inhibits mTORC1. This dephosphorylation leads to sustained mTORC1 activation, preventing autophagy initiation and aiding bacterial survival by avoiding degradation within the host cell.

Another mechanism involves Mtb’s manipulation of the mammalian target of rapamycin complex 1 (mTORC1), which is a key regulator of autophagy [10]. In addition to inhibiting phagosome maturation, the Mtb effector protein PtpA also contributes to autophagy suppression. PtpA dephosphorylates tuberous sclerosis complex 2 (TSC2), resulting in the prolonged activation of mTORC1 [10]. This sustained activation inhibits the initiation of autophagy and ensures bacterial survival while enhancing the intracellular nutrient environment that supports Mtb growth. The dynamic regulation of mTORC1 by Mtb allows the bacteria to prioritize replication during early infection stages and maintain a balanced nutrient environment in later stages (Figure 2) [10].

Mtb also secretes the zinc metalloprotease Zmp1, which blocks NLRP3 inflammasome activation, a process that normally enhances autophagy [31,32,33]. Additionally, Zmp1 interacts with mitochondrial membranes, suppressing mitochondrial ROS production and further reducing autophagy induction [31,32,33].

The actin cytoskeleton, which plays a crucial role in autophagosome membrane formation, is another target for Mtb. The secreted protein LpqN interferes with actin polymerization by targeting host actin-regulating proteins like Arp2/3, thereby blocking the early stages of autophagy [34]. This interference hampers the host’s ability to mount an effective immune response against Mtb [34].

Mtb also evades xenophagy, a selective form of autophagy that targets cytoplasmic pathogens. In addition to blocking phagosome maturation, the bacterial effector protein PknG manipulates host ubiquitin signaling, preventing Mtb from being recognized and degraded [17,23,35,36]. Recent studies suggest that PknG also inhibits LC3-associated phagocytosis (LAP), a process critical for capturing escaped bacteria within cytosolic compartments [2,17,23,35,36]. In both phagosome maturation and autophagy, Mtb also secretes CpsA to suppress ROS production, thereby limiting the oxidative burst required for effective xenophagy [36].

Mtb further exploits the balance between autophagy and apoptosis, shifting the host response from autophagy to apoptosis, which is less effective at clearing intracellular bacteria. Proteins like SecA2 play a key role in this shift, promoting bacterial survival while contributing to tissue damage and the progression of disease [37,38].

### 2.4. Manipulation of Host Metabolism

Recent breakthroughs have highlighted how Mtb reprograms host metabolism, particularly lipid pathways, to create a nutrient-rich, immunosuppressive environment that supports bacterial survival and replication [39,40,41]. Mtb specifically targets lipid metabolism to not only sustain its own growth but also to manipulate the host immune response, contributing to its broader immune evasion strategies.

One key adaptation is the induction of lipid-laden foamy macrophages, a hallmark of Mtb persistence. Studies show that Mtb secretes proteins that activate sterol regulatory element-binding proteins (SREBPs), which are crucial for regulating lipid synthesis. This activation enhances the expression of genes such as fatty acid synthase (FASN) and acetyl-CoA carboxylase (ACC), driving the synthesis of triglycerides and cholesterol [40].

Mtb also inhibits AMP-activated protein kinase (AMPK), which typically prevents excessive lipid accumulation and promotes lipophagy [42]. This dual modulation ensures the accumulation of lipid droplets, which serve as nutrient reservoirs for Mtb. Moreover, Mtb stabilizes these lipid stores by disrupting host pathways responsible for lipid droplet degradation, securing a continuous energy supply during both active and latent stages of infection [42].

The lipid-rich environment in foamy macrophages directly contributes to immune evasion [43,44,45,46,47]. Lipid-loaded macrophages exhibit reduced antimicrobial responses and the diminished production of pro-inflammatory cytokines.

## 3. Granuloma Dynamics: New Perspectives

Recent advancements in TB research have unveiled novel insights into the intricate host–pathogen interactions driving granuloma formation and persistence, highlighting sophisticated mechanisms by which Mtb manipulates the host immune system [48,49,50]. Central to these interactions are Mtb-derived lipids, including PDIMs and phenolic glycolipids (PGLs), which modulate the granuloma microenvironment by masking bacterial surface antigens [48,49,50]. These lipids prevent effective immune recognition and impair the recruitment of protective immune cells. By interfering with Toll-like receptor (TLR) signaling, PDIMs and PGLs suppress pro-inflammatory cytokine production, impair antigen presentation, and reduce macrophage activation [48,49,50]. PDIMs further enhance the survival of infected macrophages by inhibiting apoptosis, maintaining a protected intracellular niche for bacterial persistence [48,49,50].

Trehalose dimycolate (TDM), commonly known as the cord factor, plays a dual role in granuloma formation. TDM actively recruits macrophages and neutrophils, promoting granuloma maturation [51,52,53]. However, it simultaneously suppresses the bactericidal activity of these immune cells, enabling bacterial survival. Granulomas enriched with TDM are prone to necrosis, creating an environment conducive to bacterial dissemination [51,52,53]. Mycolic acids, another Mtb-derived lipid, influence granuloma structure by promoting fibrotic responses that restrict immune cell access to bacterial reservoirs, supporting chronic infection [51,52,53].

Cytokine dysregulation within granulomas also plays a pivotal role in Mtb persistence [53,54]. Elevated levels of the immunosuppressive cytokine IL-10, frequently observed in granulomas, suppress macrophage activation, dendritic cell function, and T-cell recruitment [53,54]. IL-10 reduces the production of IFN-γ by Th1 cells, a cytokine essential for macrophage-mediated bacterial killing, creating an immunosuppressive environment that enables unchecked bacterial replication [53,54]. Furthermore, Mtb induces type I interferons, such as IFN-β, which disrupt protective Th1 responses and promote the infiltration of regulatory T cells (Tregs) [53,54]. These Tregs further suppress local immunity, destabilizing granulomas and facilitating bacterial persistence.

In addition to cytokine modulation, Mtb has been found to exploit host signaling pathways to establish a favorable environment for survival. Recent studies have shown that the bacterium upregulates PPM1A, a phosphatase that inhibits apoptosis in infected macrophages [55,56,57]. By preventing programmed cell death, PPM1A allows Mtb to persist in a protected intracellular niche, evading immune detection and clearance [55,56,57]. This represents a novel target for host-directed therapies aimed at restoring macrophage apoptosis to limit bacterial survival.

Hypoxic conditions within granulomas introduce another layer of complexity. Hypoxia stabilizes hypoxia-inducible factor 1-alpha (HIF-1α) in macrophages, promoting glycolysis and bacterial dormancy while limiting immune-mediated killing [58,59]. Nitric oxide (NO) production is upregulated under hypoxia, exerting antimicrobial effects but also contributing to tissue damage and granuloma necrosis. Recent findings have identified specific neutrophil phenotypes recruited under hypoxic conditions that suppress inflammation, creating a paradoxical balance that supports bacterial survival while mitigating excessive immune activation [58,59].

Mtb also modulates macrophage activity and polarization within granulomas. By reprogramming macrophage metabolism, the bacteria drive these cells toward a glycolytic, anti-inflammatory state, reducing their capacity for pathogen killing [60]. Lipid-rich foamy macrophages, commonly found within granulomas, serve as reservoirs for Mtb, providing essential nutrients such as cholesterol to sustain bacterial growth. Additionally, infected macrophages release extracellular vesicles (EVs) carrying bacterial antigens and immunomodulatory molecules, including IL-10 and TGF-β [61,62,63]. These EVs influence nearby immune cells, further dampening immune responses and contributing to granuloma persistence.

Recent studies have highlighted the critical role of epigenetic regulation within granulomas [64,65,66]. Hypoxia-induced DNA methylation changes in Mtb drive the expression of dormancy-associated genes, enabling the bacteria to survive in latent states and resist antibiotic treatment [64,65,66]. This adaptation underscores the importance of addressing latent infections in TB management.

These findings underscore the complexity of granuloma formation and maintenance, revealing multiple novel therapeutic opportunities. Targeting Mtb-derived lipids, such as PDIMs and PGLs, to enhance immune recognition, inhibiting PPM1A to restore macrophage apoptosis, and modulating IL-10 and type I interferon pathways to rebalance immune responses are promising approaches [48,49,50]. Additionally, therapeutic interventions aimed at reprogramming macrophage metabolism or disrupting hypoxia-induced bacterial dormancy may offer effective strategies to improve bacterial clearance. Understanding these sophisticated interactions is essential for the development of therapies to address latent infections and prevent disease reactivation.

## 4. Innovative Therapies

Recent therapeutic advances provide promising approaches to combat TB by targeting the complex interactions between Mtb and the host immune system. These innovative strategies aim to enhance host defenses, disrupt immune evasion mechanisms, and modulate the granuloma environment, thereby improving bacterial clearance and reducing disease progression (Table 2).

### 4.1. Host-Directed Therapies (HDTs)

Host-directed therapies focus on modifying the host’s immune responses to enhance bacterial clearance and mitigate tissue damage caused by chronic inflammation. Several key strategies have emerged:

Autophagy Modulators: Drugs such as rapamycin and metformin have shown promise in inducing autophagy, enhancing the host’s ability to degrade intracellular Mtb [67,68,69]. Rapamycin regulates the mTOR pathway, while metformin promotes mitochondrial ROS production and AMPK activation, synergistically improving autophagic flux.

Glutathione Supplementation: Glutathione (GSH), a critical antioxidant and immune regulator, has emerged as a novel adjunct therapy [70,71,72]. GSH enhances macrophage activity, promotes bacterial killing, and modulates oxidative stress responses critical for controlling Mtb infection. Studies suggest that GSH reduces Mtb-induced inflammation, boosts T-cell proliferation, and supports immune reprogramming to favor Th1 responses [70,71,72]. Additionally, GSH helps maintain cellular redox homeostasis, which is vital for effective immune signaling during chronic infection [70,71,72].

Checkpoint Inhibitors: Immune checkpoint molecules such as PD-1/PD-L1 and CTLA-4 are manipulated by Mtb to suppress T-cell responses [73,74,75]. Targeting these checkpoints has shown promise in restoring T-cell-mediated immunity and enhancing bacterial clearance [73,74,75].

### 4.2. Cytokine-Based Immunotherapies

Immunotherapies, including recombinant IL-2, IL-7, IL-12, IL-15, IL-24, and IFN-γ, enhance immune responses, improving bacterial clearance and treatment outcomes [71]. IL-2 and IFN-γ therapies have shown improved sputum conversion rates and chest radiograph outcomes in TB patients [71]. Preclinical studies highlight IL-12’s ability to reduce Mtb loads, IL-7 and IL-15’s role in lowering bacterial burdens and enhancing survival, and IL-24’s activation of CD8+ T-cells to produce IFN-γ [71]. TLR agonists like imiquimod further boost immune responses by promoting autophagy and Th1 differentiation [71]. Anti-IL-4 antibodies shift immunity toward a Th1 profile, enhancing macrophage activity. Additionally, corticosteroids such as dexamethasone reduce inflammation and mortality in TBM, suggesting potential benefits for TB management [71].

### 4.3. Epigenetic Modulation

Mtb leverages host epigenetic mechanisms to suppress immune responses, enabling its persistence and immune evasion. Recent advances highlight how targeting these pathways through epigenetic interventions can restore effective immune defenses against TB.

HDAC Inhibitors: Histone deacetylase (HDAC) inhibitors, such as vorinostat, panobinostat, and newer compounds like entinostat, have demonstrated potential in reversing Mtb-induced immune suppression [76]. These drugs enhance the expression of immune response genes involved in pathogen recognition, autophagy, and macrophage activation. Recent preclinical studies reveal that HDAC inhibitors can synergize with first-line anti-TB drugs, improving bacterial clearance and reducing treatment duration by reactivating host antimicrobial pathways [88,89].

DNA Methylation Modifiers: Mtb induces the hypermethylation of immune-related gene promoters, silencing critical pathways for pathogen control [77,90]. Agents targeting DNA methyltransferases (DNMTs), such as decitabine, are being investigated for their ability to reverse this silencing [77,90]. Restoring the gene expression of key cytokines like IFN-γ and chemokines critical for T-cell recruitment has shown promise in addressing both active and latent TB.

MicroRNA-Based Modulation: Mtb also manipulates host microRNAs (miRNAs) to suppress immune signaling. For instance, the upregulation of miR-155 and miR-146a has been linked to impaired macrophage function [78,79]. Recent findings propose that anti-miRNA therapies, which inhibit Mtb-induced miRNA expression, can restore cytokine production and enhance bacterial killing [91].

Histone Modifications: Mtb alters histone acetylation and methylation patterns to modulate chromatin structure, affecting immune response gene accessibility. Drugs targeting bromodomain and extraterminal (BET) proteins, such as JQ1, are emerging as potential therapeutic options [80,81]. BET inhibitors have shown efficacy in promoting anti-inflammatory responses and enhancing host defense mechanisms in preclinical TB models [80,81].

Long non-coding RNAs (lncRNAs): Recent research highlights the role of lncRNAs in regulating immune responses during Mtb infection. Targeting lncRNAs that suppress immune activation, such as lncRNA MEG3, has been proposed as a novel approach to enhance host resistance [82,83,84].

These epigenetic strategies, when combined with conventional anti-TB therapies, hold promise for improving treatment outcomes, shortening therapy duration, and addressing latent and drug-resistant TB. Advances in epigenetic profiling and targeted delivery systems are further accelerating the development of these innovative interventions.

### 4.4. Targeting Granuloma Dynamics

Granulomas, complex immune structures central to TB pathogenesis, serve as both protective barriers and reservoirs for Mtb. Targeting granuloma dynamics to enhance immune responses while limiting bacterial persistence and tissue damage is a growing area of TB therapy research. Recent findings have expanded our understanding of therapeutic interventions.

Matrix Metalloproteinase (MMP) Inhibitors: MMPs, particularly MMP-1 and MMP-9, play a critical role in granuloma remodeling, contributing to extracellular matrix degradation, tissue destruction, and granuloma necrosis [92]. Inhibitors such as doxycycline have demonstrated efficacy in reducing MMP activity, preserving granuloma structure, and preventing bacterial dissemination. Recent studies suggest that combining MMP inhibitors with standard anti-TB therapy may enhance treatment outcomes by reducing lung cavitation and improving bacterial clearance [85].

Anti-Angiogenic Therapies: Granulomas require vascular remodeling to sustain their structure, but excessive angiogenesis can create hypoxic environments that promote Mtb persistence [86]. Drugs like bevacizumab, an anti-VEGF monoclonal antibody, disrupt granuloma vasculature, limiting the nutrient and oxygen supply essential for Mtb survival [93]. Recent studies show that anti-angiogenic therapies reduce bacterial burdens and enhance immune accessibility to granuloma cores [94,95]. Emerging research is exploring combination therapies that pair anti-angiogenic agents with oxygen delivery systems to counteract hypoxia-induced dormancy.

Hypoxia Modulation: Hypoxia within granulomas stabilizes HIF-1α, which shifts macrophages toward glycolytic metabolism, supporting bacterial persistence. Recent findings highlight the potential of HIF-1α inhibitors or arginine supplementation to modulate hypoxia and restore immune function [58,87]. These approaches not only enhance macrophage bactericidal activity but also prevent granuloma necrosis and limit bacterial dissemination.

Fibrosis Control: Excessive fibrosis in granulomas can shield Mtb from immune attack while contributing to chronic lung damage. Therapies targeting fibrosis, such as the inhibitors of transforming growth factor-beta (TGF-β) signaling, are under investigation for their ability to maintain granuloma stability while improving immune cell penetration [53,72].

These approaches underscore the importance of modulating granuloma dynamics to balance protective immune responses with minimal tissue damage. Advances in the imaging and molecular profiling of granulomas are facilitating the development of targeted therapies to disrupt Mtb niches while preserving host tissue integrity.

## 5. Future Directions and Challenges

The dynamic interplay between Mtb and the host immune system presents a complex landscape for therapeutic interventions. While recent advances in molecular biology, immunology, and bioinformatics have provided unprecedented insights into host–pathogen interactions, several challenges and opportunities lie ahead in the fight against TB.

### 5.1. Addressing Latent TB and Drug Resistance

Latent TB, a condition where Mtb persists in a dormant state within granulomas, continues to pose a significant challenge. Despite advancements in understanding granuloma biology and the molecular pathways that drive Mtb dormancy, effective treatments for latent TB remain elusive. Future research must focus on targeting hypoxia-driven bacterial dormancy mechanisms while preserving granuloma integrity to minimize tissue damage. The emergence of multidrug-resistant (MDR) and extensively drug-resistant (XDR) TB underscores the urgency for new therapeutic strategies [96,97]. HDTs, epigenetic interventions, and novel immunomodulatory agents show promise, but their efficacy in clinical settings needs validation. Advances in high-throughput drug screening, CRISPR-based gene editing, and systems biology can accelerate the identification of effective HDTs tailored to combat drug-resistant TB strains [98,99,100,101,102].

### 5.2. Integration of Advanced Technologies

The integration of artificial intelligence (AI) and machine learning (ML) in TB research offers exciting opportunities to analyze complex datasets, identify biomarkers, and predict treatment outcomes [103,104,105]. Multi-omics approaches, including transcriptomics, proteomics, metabolomics, and lipidomics, are crucial for mapping the intricate host–pathogen interactions [106,107,108]. These tools can guide the design of targeted therapies aimed at disrupting bacterial niches without compromising host immunity.

### 5.3. Vaccine Development

Despite the widespread use of the Bacillus Calmette-Guérin (BCG) vaccine, its efficacy in preventing pulmonary TB in adults remains limited. Recent advances in vaccine research, including subunit vaccines, viral vector-based platforms, and mRNA vaccines, offer promising alternatives [71]. Addressing the variability in vaccine responses across populations, particularly in high-burden regions, will require personalized approaches based on genetic, environmental, and microbiome-related factors.

## 6. Conclusions

TB remains a formidable global health challenge, necessitating a multidisciplinary approach to understand and combat the disease. Recent advances in elucidating host–pathogen interactions have paved the way for innovative therapeutic and preventive strategies. The exploration of immune evasion mechanisms, granuloma dynamics, and host-directed therapies highlights the potential to enhance treatment outcomes and address latent infections.

However, significant challenges persist, including the need for effective vaccines, the rise in drug-resistant TB strains, and the equitable distribution of emerging therapies. Future research must leverage advanced technologies, such as AI, multi-omics, and high-resolution imaging, to unravel the complexities of TB pathogenesis. Collaboration across scientific disciplines, global partnerships, and sustained investment in TB research and healthcare infrastructure will be vital to achieving the long-term goal of TB eradication.

## Figures and Tables

**Table 1:** Mechanisms employed by Mtb to inhibit autophagy.

Mtb Effector/Protein	Mechanism of Action	Impact on Autophagy	References
PDIM	Inhibits LC3-associated phagocytosis (LAP) by blocking the phagosome recruitment of NADPH oxidase.	PDIM protects Mtb from LAP and classical autophagy, helping the bacteria survive in non-alveolar macrophages in an autophagy-dependent manner.	[26]
EsxH	Interferes with Endosomal Sorting Complex Required for Transport (ESCRT) machinery by targeting the VPS4 subunit.	Prevents autophagosome–lysosome fusion, halting autophagy and allowing Mtb to evade degradation within macrophages.	[27]
EsxA (ESAT-6)	Forms pores in the lysosomal membrane, causing dysfunction. Activates the cGAS-STING pathway.	Disrupts lysosomal acidification, inhibits autophagic degradation, and increases type I interferon production, which suppresses autophagy.	[28,29,30]
PtpA	Dephosphorylates TSC2, leading to prolonged activation of mTORC1.	Sustained mTORC1 activation inhibits autophagy initiation, promoting bacterial survival and supporting intracellular growth.	[10]
Zmp1	Interacts with mitochondrial membranes and blocks NLRP3 inflammasome activation.	Reduces mitochondrial ROS production, further hindering autophagy induction.	[31,32,33]
LpqN	Interferes with actin polymerization by targeting host actin-regulating proteins like Arp2/3.	Disrupts early stages of autophagy by blocking autophagosome membrane formation.	[34]
PknG	Manipulates host ubiquitin signaling.	Prevents Mtb from being recognized and degraded by autophagy. Inhibits LC3-associated phagocytosis (LAP), which captures cytosolic bacteria.	[17,23,35,36]
CpsA	Suppresses ROS production by inhibiting NADPH oxidase activity.	Limits the oxidative burst required for effective xenophagy (selective autophagy of cytoplasmic pathogens).	[17,23,35,36]
SecA2	Modulates host response by shifting from autophagy to apoptosis.	Induces apoptosis, which is less effective at clearing intracellular bacteria compared to autophagy.	[37,38]

**Table 2:** Innovative therapies targeting host–pathogen interactions in TB.

Therapy Category	Therapy/Agent	Mechanism of Action	Targeted Mtb Mechanism	Impact on TB Treatment	References
Host-Directed Therapies.	Autophagy Modulators	Rapamycin (mTOR pathway modulator) and metformin (AMPK activator) enhance autophagy to degrade Mtb.	Blocks Mtb’s evasion of autophagy.	Promotes bacterial clearance, reduces intracellular survival of Mtb.	[67,68,69]
	Glutathione Supplementation	Enhances macrophage activity, reduces Mtb-induced inflammation, and supports Th1 immune responses.	Modulates oxidative stress, boosts immune reprogramming for Th1 responses.	Enhances T-cell proliferation, promotes bacterial killing, supports chronic infection control.	[70,71,72]
	Checkpoint Inhibitors	Inhibits immune checkpoint molecules (PD-1/PD-L1, CTLA-4) to restore T-cell responses.	Mtb manipulates checkpoint molecules to suppress immunity.	Restores T-cell immunity, enhances bacterial clearance.	[73,74,75]
Cytokine-Based Immunotherapies	Recombinant Cytokines	IL-2, IL-7, IL-12, IL-15, IL-24, and IFN-γ therapies enhance immune responses to clear Mtb.	Boosts immune responses, enhances macrophage and T-cell activation.	Improves sputum conversion, reduces bacterial load, activates CD8+ T-cells.	[71]
	TLR Agonists	Imiquimod activates Toll-like receptors to promote autophagy and Th1 differentiation.	Stimulates immune pathways to enhance antigen presentation.	Boosts immune responses, enhances autophagic activity, and improves immune function.	[71]
	Anti-IL-4 Antibodies	Shifts immune response from Th2 to Th1, enhancing macrophage activity.	Mtb manipulation of cytokine balance (Th1 vs. Th2).	Increases bacterial clearance by promoting Th1 immunity.	[71]
	Corticosteroids	Dexamethasone reduces inflammation, potentially reducing TBM-related mortality.	Modulates immune responses and inflammation.	Potential to reduce inflammation, enhance treatment efficacy in TBM.	[71]
Epigenetic Modulation	HDAC Inhibitors	Vorinostat, panobinostat, entinostat restore immune responses by reactivating immune genes.	Reverses Mtb-induced immune suppression by modulating histone acetylation.	Enhances pathogen recognition, improves bacterial clearance, shortens treatment.	[76]
	DNA Methylation Modifiers	Decitabine inhibits DNA methyltransferases, reversing immune suppression through DNA demethylation.	Silences key immune-related gene promoters.	Restores gene expression of immune regulators like IFN-γ, improves immune response in latent and active TB.	[77]
	MicroRNA-Based Modulation	Anti-miRNA therapies block Mtb-induced miRNAs, restoring cytokine production and enhancing bacterial killing.	Suppression of miRNA expression impairs immune signaling.	Improves immune responses, enhances bacterial killing, restores macrophage function.	[78,79]
	Histone Modifications (BET Inhibitors)	JQ1 inhibits BET proteins, modulating chromatin structure and immune responses.	Mtb alters histone acetylation and methylation patterns.	Enhances anti-inflammatory responses, improves immune activation.	[80,81]
	Long Non-Coding RNAs (lncRNAs)	Targets lncRNAs like MEG3 to enhance immune activation.	Mtb modulates lncRNA to suppress immune responses.	Promotes enhanced resistance to TB by improving immune activation.	[82,83,84]
Targeting Granuloma Dynamics	Matrix Metalloproteinase (MMP) Inhibitors	Doxycycline reduces MMP activity, preserving granuloma structure and preventing bacterial dissemination.	Mtb exploits MMPs for granuloma remodeling.	Reduces tissue destruction, preserves granuloma structure, enhances bacterial clearance.	[85]
	Anti-Angiogenic Therapies	Bevacizumab (anti-VEGF) disrupts granuloma vasculature, limiting nutrient and oxygen supply to Mtb.	Mtb thrives in vascularized granulomas, promotes survival in hypoxia.	Reduces bacterial load, enhances immune access to granulomas, counters Mtb persistence.	[86]
	Hypoxia Modulation	HIF-1α inhibitors or arginine supplementation restore immune function in hypoxic granulomas.	Hypoxia stabilizes HIF-1α, aiding Mtb persistence.	Enhances macrophage bactericidal activity, reduces granuloma necrosis.	[58,87]
	Fibrosis Control	TGF-β inhibitors reduce fibrosis in granulomas, enhancing immune cell penetration.	Excessive fibrosis protects Mtb from immune attack.	Improves granuloma stability, enhances immune cell infiltration and bacterial clearance.	[53,72]

## Data Availability

Data available in references.
